# Use of digital facilitation to support the use of digital services in general practice in England: An interview study with key stakeholders

**DOI:** 10.1177/13558196251316446

**Published:** 2025-01-30

**Authors:** Bethan Mair Treadgold, Rachel Winder, Helen Atherton, Carol Bryce, John Campbell, Christine Marriott, Jenny Newbould, Stephanie Stockwell, Emma Pitchforth

**Affiliations:** 1 Research Fellow, Exeter Collaboration for Academic Primary Care, 171002 University of Exeter Medical School, Exeter, UK; 2 Professor of Primary Care Research, Unit of Academic Primary Care, Warwick Medical School, Warwick, UK; 3 Assistant Professor, Unit of Academic Primary Care, Warwick Medical School, Warwick, UK; 4 Professor of General Practice and Primary Care, Exeter Collaboration for Academic Primary Care, 171002 University of Exeter Medical School, Exeter, UK; 5 Patient and Public Contributor, 574931 NIHR Applied Research Collaboration South West Peninsula Patient Engagement Group, Exeter, UK; 6 Research Leader, 89908 RAND Europe, Cambridge, UK; 7 Senior Analyst, 89908 RAND Europe, Cambridge, UK; 8 Associate Professor, Exeter Collaboration for Academic Primary Care, 171002 University of Exeter Medical School, Exeter, UK

**Keywords:** digital services, primary care, qualitative research

## Abstract

**Objective:**

Digital services in primary care are becoming more common, yet access to and use of services can create inequities. Our aim was to explore the drivers, priorities, and evolving policy context influencing digital facilitation in primary care as reported by national, regional and local level stakeholders in England.

**Methods:**

We conducted online semi-structured qualitative interviews with stakeholders, including those in NHS England organisations, local commissioners for health care, statutory and third sector organisations, those working within the research community, and digital platform providers. Interviews were analysed using a thematic approach.

**Results:**

The majority of stakeholders worked in national level roles, in commissioning or statutory and third sector organisations working in relation to digital inclusion and patient access. Demographic inequalities, poor usability of digital primary care services, and low digital skills were perceived to comprise some of the barriers facing patients in accessing and using digital primary care services. Demand pressures in general practice, inconsistent training opportunities in digital services for staff, and conflicting perceptions around who should be responsible in organising digital facilitation were reported as barriers in the organisation and provision of digital facilitation in primary care. Stakeholders shared future visions for digital primary care and recommended focusing on establishing the concept of digital facilitation and promoting the benefits in its adoption.

**Conclusions:**

Policy that is specific to digital facilitation and not just to digital services is required to establish clear lines of responsibility, investment in staff time and training, and the development of digital services that work well for various groups of patients and practice staff. A multi-organisational working team involving decision-makers and those working on the ground in general practice is encouraged to establish principles for supporting patients and staff in accessing and using digital primary care services in the NHS in England.

## Introduction

There has been a move towards the adoption of digital services in primary care in many countries. For example, New Zealand and Australia have increased specific commitment and funding to digital services in primary care, partially in response to COVID-19^1,2^ while Germany and Estonia have been noted for their rapid digitisation of the health system more broadly.^3,4^ In England, a range of digital services has been made available, with contractual requirements for general practitioners (GPs) to offer such services,^4^ including booking an appointment, ordering a repeat prescription, viewing patient records, checking symptoms, updating personal details, and remote consultations (e.g., email, telephone, online messaging, and video).^5^ This has been part of a wider move to promote digital services across health and social care in an effort to transform public services.^6^

The drivers of digital services in primary care include the convenience for people to access general practice, improved streamlining and triaging of patient requests in a timely manner and the ubiquity of digital services in everyday life for patients and staff.^7^ The COVID-19 pandemic has accelerated the shift to remote consulting and reliance on digital resources in general practice,^8^ with 85% of primary care consultations taking place remotely at the height of the pandemic^9^; this has since reduced, with remote consultations accounting for around 30% of primary care appointments in 2023.^10^ The same year, 32 million people were reported to have downloaded the NHS app, which can facilitate access to a patient’s health record and to booking appointments, and 28 million people had acquired an NHS login.^11^

The expansion of digital services in primary care raises important questions about equity in access. In 2022, the use of digital primary care services varied widely across England, from 46% of patients in some areas to a maximum of 70% in other areas.^12^ Patient-reported barriers to digital service use include lack of experience of using the internet and, thus, digital services,^13,14^ lower health literacy,^13,15^ low usability of digital services,^16,17^ and lack of motivation or understanding of the use and usefulness of such services.^18–20^ Digital inequalities tend to adversely affect older people, non-white ethnicities, those in lower socioeconomic groups, those in poorer health including living with mental illness, and people in rural settings.^13,21–25^ There is commitment to address digital exclusion,^24–26^ but investment in addressing gaps in the uptake and use of digital services offered by the National Health Service (NHS) has remained limited.^27^

‘Digital facilitation’, defined as “that range of processes, procedures, and personnel which seeks to support NHS patients in their uptake and use of digital services”^28^ ^(p. 5)^ can provide an important means to enhance patient and carer access to and use of digital services. Digital facilitation may include promotion, skills development, guidance, and support, although more evidence is needed about the effectiveness of different approaches for populations most in need.^29–33^ There are currently no specific policies concerning the roles, responsibilities and mechanisms in and for digital facilitation. Variation in digital platforms used in primary care locally alongside nationally provided services such as the NHS app means that different forms of support may be needed.

This study aimed to understand the drivers, priorities, and evolving policy context influencing digital facilitation in primary care as reported by a range of local, regional and national level stakeholders with an interest in, or likely influence on, decision-making on or provision of digital facilitation in England. Part of a larger research project (The Di-Facto study),^28^ we draw on focused ethnographic research and surveys in general practices, incorporating the views and experiences of patients, carers and staff.^19,20,32,33^ Our study contributes to the understanding of the wider policy context in which digital facilitation can be understood.

## Method

This was a qualitative study using semi-structured interviews. Reporting of this study was guided by the Standards for Reporting Qualitative Research^34^ (Online Supplement). We drew on expert interview methodology, which is well suited to a relatively new field.^35–37^ The approach enables access to a range of views and expert knowledge as derived from practical experiences and people’s place within institutions.

### Sampling and recruitment

We sought to recruit 12-18 stakeholders, defined as individuals who may have critical oversight or involvement working at local, regional, and national levels in England on matters relating to digital facilitation in primary care. We conducted an initial stakeholder analysis, which involved contact mapping using policy review, the research team’s knowledge of the health system and patient and professional bodies, professional networks of the wider research team, and internet searches. We then used snowball sampling^38^ to identify further stakeholders. Table 1 shows the categories of stakeholders targeted, including their assumed role or interest in digital facilitation. Identified stakeholders were invited for an interview by email, which included an information leaflet about the study. Stakeholders could suggest an alternative appropriate contact.

**Table 1.** Targeted stakeholders in digital facilitation policy.

Category of stakeholder	Assumed role or interest in digital facilitation	Description of stakeholders targeted
National and regional NHS bodies	Digital facilitation would have direct relevance for meeting broader NHS policy goals^9^ and NHS action on digital inclusion.^43^	Persons working within the NHS infrastructure around digital or primary care related matters
Organisations responsible for the commissioning or organisation of health and care services locally	Digital facilitation is relevant in the context of commissioning digital services and meeting the needs of local population, including in relation to digital inclusion.^43^	Clinical commissioning groups Newly forming integrated care systems Primary care networks
Statutory and third sector organisations working in digital inclusion or patient access	Nationally and locally third sector organisations and some statutory organisations have a dedicated remit to improve digital inclusion or to represent patients. NHS England digital inclusion policy has drawn on work of third sector organisations	National, regional and local charities dedicated to supporting groups of the population who typically experience digital exclusion such as the elderly, ethnic minority groups, those experiencing homelessness, refugees and those living with long-term health conditions
Digital platform providers	Assumed relevance for digital facilitation as either providers of support or with an interest in efforts to support patients and carers to use online services	Providers of primary care digital consultation and triaging platforms
Research community relevant to digital access and online primary care services	To incorporate different professional insight and ongoing research into digital facilitation at conceptual or practical level	Individuals identified through existing networks and published research

### Data collection and procedure

Semi-structured qualitative interviews were conducted by BT and RW using an interview topic guide (Online Supplement), which was informed by the research questions, existing evidence and previous studies conducted by co-investigators, and refined in light of findings from the other elements of the Di-Facto research study. The topic guide explored: (i) the key drivers of digital facilitation; (ii) how stakeholders thought digital facilitation worked and the intended consequences of its application; (iii) concerns around digital inclusion; and (iv) the evolving policy context that the study could help inform. Prompts were included to encourage further elaboration as necessary. The concept of ‘digital facilitation’ was introduced at the beginning of each interview and participants were invited to discuss the term.

Interviews were conducted online using video conferencing software (Microsoft Teams, Google Hangouts and Zoom) between October 2021 and May 2022. Consent was obtained via an online form before all interviews, and with permission, interviews were audio-recorded. Study participants were assured that all potentially identifiable information (e.g., names, locations, organisations) would be removed from quotations in publications of the study. Interview length ranged from 22 to 62 minutes.

### Data analysis

Audio recorded interviews were transcribed using a professional transcription company and transcripts checked for accuracy by BT and RW. Interview data were analysed using a systematic qualitative thematic analysis and following a mixed inductive-deductive reflexive approach, which involved six stages^39,40^: (1) all interview transcripts were read by at least one researcher; (2) three researchers (BT, RW, EP) coded the transcript, with an initial coding frame developed while coding was ongoing until saturation was achieved; (3) the coding frame was further developed and iterated with the whole research team (BT, RW, HA, CB, JN, SS, EP) including a patient and public contributor (CM), alongside qualitative data from the wider Di-Facto study; (4) all authors wrote a one-page summary of a code each, then further explored the coded data for overarching thematic topics and wrote one-page summaries of overarching themes; (5) thematic summaries were discussed and iteratively refined at team meetings along with JC, and the final overarching themes were developed; (6) findings were written up by BT.

### Patient and public involvement

CM was member of a dedicated patient and public involvement group to the wider Di-Facto research study, and contributed during the analysis stage of the study. CM read a selection of the one-page summaries written by the research team and contributed to the development of the coding frame and interpretation of the data.

### Ethical approval

Ethical approval was granted by the Newcastle & North Tyneside 2 Research Ethics Committee (reference number: 21/NE/0079) in April 2021.

## Results

Nineteen stakeholders participated in the study (Table 2), representing a response rate of 32% of an initial sample of 59 stakeholders who were invited to take part. The majority of participants worked in commissioning roles (*n* = 6) or in statutory and third sector organisations (*n* = 6). The majority worked in national level roles (*n* = 11). Ten stakeholders maintained clinical roles in primary care while holding senior positions working on matters relating to digital inclusion and primary care. Of those not taking part, 26 declined and 22 did not respond to our invitation; they all worked either nationally or regionally within NHS England organisations or in statutory and third sector organisations.

**Table 2.** Characteristics of stakeholders in terms of organisation type, level, and clinical or non-clinical status.

Characteristics of stakeholders	*N* = 19
Organisation type	*n*
Commissioning or organisation of care to meet local population needs	6
Statutory and third sector organisations	6
NHS bodies	4
Research community	2
Digital platform providers	1
Level	
National	11
Regional	5
Local	3
Clinical/non-clinical	
Clinical role	10
Non-clinical role	9

Our analysis developed three overarching themes: (1) digital services in primary care are not necessarily accessible, usable, or advertised widely for all groups of patients; (2) the over-burdened nature and context of general practice can impedes digital facilitation; and (3) suggestions for moving digital facilitation in primary care forward. We report on each in turn.

### Digital services in primary care are not necessarily accessible, usable, or advertised widely for all groups of patients

Discussion of digital facilitation prompted stakeholders to reflect on factors which may prevent people from benefiting from digital primary care services. Identified barriers included demographic inequalities, poor usability of digital services, and lack of awareness of and confidence in using these services. These barriers were thought to further disadvantage those who are already at risk of digital exclusion in primary care. Stakeholders noted that such barriers do not appear to be reduced by current policy, despite existing commitments to address them.

#### Demographic inequalities

Stakeholders described a range of groups to be at risk of being excluded from digital access and support, including people who do not speak or read English, older people, those with cognitive disabilities, and those in precarious living conditions. Some stakeholders noted that most health care and support materials are provided in English but that translation services or other accessibility services for those with learning disabilities, hearing or sight impairments are not well suited to digital consultations and apps.

Several stakeholders identified people experiencing homelessness, traveller communities, sex workers, and refugees also to be at risk of being excluded from digital services and support. While having varied needs and experiences of digital services in primary care, common reasons for not accessing such services included lack of trust regarding privacy and security, and of having a trusted person such as a family member or third party to help them access and use digital services. These groups would also not have regular access to a computer or smart phone, the internet, or an email address, which is often required to access the services.

*So a lot of the homeless hotels, for example, set up through the pandemic didn’t have free WiFi. So, although there was WiFi available, you might give somebody a device you’d still need to pay for them to get WiFi access in order to have any kind of contact. So, all of that puts other barriers in place*. (Stakeholder A, Third sector organisation, national level, with clinical role)

#### Poor usability of primary care systems or technology

Stakeholders working in third sector organisations and clinicians frequently discussed the challenges posed by differences in the usability of digital platforms. Platforms for ordering repeat prescriptions were described as easy to use, while certain digital consultation platforms that include a form-based online consultation and triage platform were described as ‘tedious’. These were seen to result in users giving up or they would instruct users to make an appointment with their GP for a matter that could be dealt with elsewhere.

The process for registering patients with digital platforms was also described as confusing.

*So the letter was saying things like, it would say, ‘Patient ID,’ and then have a string of numbers for patient ID so, so they could log in, but the, online when you were registering it would say ‘Access ID.’ Now, both those numbers were exactly the same but the patients were going online and seeing ‘Access ID,’ but they have ‘Patient ID’ in front of them. That, that was it, they would stop, they, they would hit a, they would hit a stumbling block, they would hit a brick wall and they would stop*. (Stakeholder Z, Third sector organisation, regional level)

Stakeholders highlighted issues around platform interoperability such as delays in test results being uploaded to patients’ digital platforms, and availability of timeslots to book appointments not being up-to-date. Some study participants felt that it may be challenging for patients to navigate the range of different platforms and services used within a single GP practice, and that it may be frustrating when these platforms and interfaces appear dated in comparison to what patients experience elsewhere, such as when using online banking and shopping.

#### Lack of awareness of digital services available and confidence using them

There were mixed views on perceived patients’ digital skills and confidence. Some participants thought that people’s skills and awareness of digital services had improved during and since the COVID-19 pandemic, with remote consultations and other aspects of living such as connecting with friends and family becoming increasingly digitalised.

However, despite these developments, many stakeholders thought that generally, adults often lacked essential digital skills and the confidence to navigate the evolving digital world. This was seen to act as a barrier in accessing digital primary care services.

*[A]bout 8.7 million adults who might have the essential digital skills for life but that’s not enough, they don’t yet meet the standard for what’s needed for working life to take it into the work place. So, across, across the board, across all ages you're talking about a lack of digital skills at that really essential level.* (Stakeholder M, Third sector organisation, national level)

### The over-burdened nature and context of general practice can impede digital facilitation

Participants highlighted ‘on the ground’ issues in the day-to-day workings of general practice, which present as barriers to digital facilitation. These included demand pressures, conflicting perceptions of roles and responsibilities for digital facilitation, poor usability of systems, staff attitudes and experience, and lack of training. These issues were not currently being addressed by policy, preventing some patients and staff from benefiting from digital primary care services.

#### Demand pressures in general practice

Participants working in primary care noted that general practice staff thought that increased digital access would lead to increased demand. One stakeholder described how some practices would reduce or stop digital services because of concerns about higher demand (*‘Well we’re not coping so we’ve had to switch off the digital thing’ *(Stakeholder W, NHS organisation, regional level, with clinical role)). Some pointed to a lack of collective decision-making at practice level on digital services and provisions for digital facilitation.

*[A] lot of these decisions are being made by GP partners, and a lot of GP, or senior GPs and a lot of the GP partners, senior GPs are, you know, technophobic for want of a better word. And they… just don’t like using technology*. (Stakeholder B, Academia, with clinical role)

#### Conflicting perceptions of roles and responsibilities for digital facilitation

There were differing views on who should organise or carry out digital facilitation. Many stakeholders thought that reception staff, as the first point of call for patients were driving digital facilitation.

*The reality is it’s probably not the GPs. Because actually by the time the patient’s got to them, you know, they’ve, they’ve got in front of the GP. So I think it, generally what we’ve found is it’s the, it’s the front line staff on the desk that… Because they’re the ones that take the initial contact from the patient… So I’d say, I don’t know if responsibility is the right word, but definitely they have the, the power to drive that change.* (Stakeholder J, Third sector organisation, national level)

Some stakeholders felt that digital facilitation should be driven from outside general practices, including the wider health care system and digital platform providers. Others noted that the NHS generally should ensure that patients can interact with health services digitally. Only one stakeholder felt strongly that digital literacy should not be the responsibility of the health care system.

*Fixing the people’s IT literacy is not the sole function or responsibility for health care. ‘Cause this is a societal problem*. (Stakeholder J, Third sector organisation, national level).

Overall, there was agreement on the need of a front-desk, multi-skilled professional with strong digital competencies.

*[W]e have a practice and a patient services [person], and he is really IT literate and does all of our bits and pieces and without him, we would struggle*. (Stakeholder P, NHS organisation, Local level, with clinical role)

#### Poor usability of primary care systems in general practice

Participants working in primary care described a lack of awareness of and communication between those developing digital services and those using these services in practice.

*[I]t really boggles my mind to, to, to understand why on one hand we’re trying to do really exciting things like digital virtual wards and yet on the other hand it seems nobody is sitting down and saying, ‘Hang on, [electronic patient records system] isn’t working properly still, is anyone going to actually sort that out?* (Stakeholder N, Regional level in clinical role)

Digital developers interviewed for this study further pointed to a series of challenges of technology on the ground, such as limited interoperability. Those working in primary care also thought that existing digital services were not developed to be people focused. As noted above, stakeholders described difficult registration processes, and that systems in place did not allow for straightforward communication and correspondence between staff and patients.

#### Staff attitudes to and limited experience of using digital services

As already noted, there were concerns about limited skills of some staff, in particular receptionists, which meant they were unable to support patients in accessing and using digital services. The rapidly evolving technology also meant that the introduction of new digital systems within short periods of time would challenge staff in primary care to operationalise and make best use of the new services.

*And then for other people, suddenly their, you know, their poor, the poor practice manager is told, “Right, you’ve come, got to implement this now.” And nobody in the practice knows how to make it work*. (Stakeholder E, Third sector organisation, national level)

Participants further highlighted that staff attitudes towards using digital primary care services could undermine use of digital primary care services, with reports of resistance among some staff to change and embrace new digital services.

*I think patients have never really been a barrier. I think clinicians are different, because I think there’s, kind of, real mixed reception from clinicians. Some clinicians totally get it and embrace it. Others are, are very resistant to change.* (Stakeholder Q, Provider of digital platform, with clinical role)

#### Training opportunities in digital facilitation

Many participants thought that existing training opportunities in digital services and facilitation were not communicated or promoted well enough across general practice, and that this needed to improve in order to grow digital provision.

*How we communicate training offers and raise awareness of what’s available needs to be improved… But I think it’s, it’s got to be bigger than just our traditional routes*. (Stakeholder K, NHS organisation, regional level, with clinical role)

Some felt that digital training programmes were communicated, but the offer and range programmes was confusing with staff often unclear which to complete. There was also a perception among some that relevant training was not prioritised in general practice as it was an activity in addition to fundamental clinical training.

A few participants highlighted the general lack of a broader policy for digital facilitation training for general practice staff although one pointed to an existing training programme, which, at the time of interviews, was in development with Health Education England (body responsible for delivery and reform of education for NHS workforce, now part of NHS England), to support practice staff in digital facilitation.

### Suggestions for moving digital facilitation in primary care forward

It was evident throughout the interviews that stakeholders were giving active thought to ‘digital facilitation’ and they identified a set of suggestions for how to improve digital facilitation within the context of an evolving primary care.

#### Establish digital facilitation as a concept in primary care

Most stakeholders valued the concept of digital facilitation. Some saw it as workable and flexible. There was agreement that digital facilitation could improve access and, thus, health care. However, there were also concerns that it would be difficult to communicate to the wider public.

*I, I don’t understand what that means, I don’t think other people would, not everyone… I wouldn’t understand what that means? … I don’t want to pigeonhole anyone, but if you said it to my mum, she would have no idea what you’re talking about*. (Stakeholder S, NHS organisation, national level)

Stakeholders suggested alternative terms for digital facilitation that may be more widely understood.

*There is something around using the word, ‘support,’ rather than facilitation and rather than digital using something like, ‘online.’ So, I think those, those are two words that probably are better understood by the general public than, than digital and facilitation*. (Stakeholder K, NHS organisation, regional level, with clinical role)

#### Examples of digital facilitation to guide future operations

Examples of digital facilitation in primary care that were discussed included community-based digital skills sessions and the provision of digital facilitation in general practice waiting rooms.

*So that involved us taking some tablets and devices and laptops out to GP waiting rooms in the [name of town] area… and just sitting down with people and just being in the corner and sort of saying, “By the way, I’m here showing people how to log on and use the online services if you want any advice,” and it went really well*. (Stakeholder Z, Third sector organisation, regional level).

Other participants, including digital platform providers, made recommendations based on their experiences of organising digital facilitation session with general practice staff, including receptionists and social prescribers.

*So certainly in our practice they’ve all got scripts to follow that, you know, are, “Well, actually, we have this new service now.” You know, “Are you able, are you able to access online,” you know, “the web, internet?” “Yes.” “Okay. We, please visit our website or you can download the NHS app…. So, and it’s, and it’s, you know, you tweak it, depending on what sort of model the practice want to follow*. (Stakeholder Q, Provider of digital platform, with clinical role).

## Discussion

This study sought to understand the views and experiences of a wide range of stakeholders involved in the commissioning or delivery of digital primary care services, regarding the drivers, priorities and evolving policies shaping support for patients in the use of such services. Overall, participants agreed about the potential for patients and practices to benefit from an expansion of digital services. However, they described a number of challenges for digital facilitation that were not being addressed by policy. Chief among these were demographic inequalities, poor usability of digital primary care services, and low digital skills and confidence. A lack of clear responsibilities, variation between digital platforms and insufficient training for staff were seen to further undermine efforts to support patients in the use of digital services. Together, our findings point to the need for greater clarity within an evolving policy context on how to best support people to access and use digital services, particularly those at greater risk of being further disadvantaged by an increasing focus on digital provision. Our findings further suggest that there is a need to better support staff and patients in the access and use of services and for this to recognise the range of resources required and challenges faced among different population groups and within general practice.

### Findings in the context of existing research

As digital facilitation is an emerging concept, it is challenging to set our findings in the context of existing research. Our earlier scoping review found that digital facilitation may be effective in promoting the uptake and use of digital services among patients.^29^ It highlighted that users’ perceptions of the usefulness of digital services, trust in the service, capacity of primary health care staff, guidelines supporting facilitation efforts, and staff motivation all affected uptake of digital primary care services. The review further showed that providing technical training for patients at risk of exclusion could be effective in reducing digital inequalities. Our study adds to these findings by highlighting the barriers to organising and delivering digital facilitation, which include conflicting views on roles and responsibilities, and lack of standardised training opportunities. Our findings are also in line with the findings of a review of qualitative literature of patients and carers’ engagement with digital health technology, which also identified usability of technology and digital literacy of as common barriers to the take up of digital health services.^16^

Our findings further align with international experience of developing and implementing digital health care strategies, which point to the need for clarity about responsibilities and accountability, and the need for involving multiple stakeholder and co-design solutions.^41^

### Strengths and limitations

We successfully recruited a broad range of stakeholders, including those working at various levels, across multiple organisations, and many with decision-making roles as well as experience of general practice. Stakeholder’s discussions of the drivers, priorities, and evolving policy context shaping digital facilitation in primary care were remarkably similar, and data saturation was deemed to have been achieved. We recruited participants at a time when those in professional roles were under extreme pressure during the COVID-19 pandemic. Conducting interviews during the COVID-19 pandemic meant that we were able to explore the increased use of, and the development of new ways of accessing, digital primary care services.

We had to approach many individuals to achieve our sample size. Possible reasons for this include a lack of perceived importance of digital facilitation, the COVID-19 pandemic, time constraints, or more broadly the challenges of engaging policymakers and other stakeholders in research. The use of online interviews is likely to have facilitated access however.^42^ We attempted to recruit stakeholders to represent a range of patient groups for whom digital facilitation may be particularly important, and while we identified several potential representatives, we were unable to recruit stakeholders with expertise in mental health. As this study was conducted during the COVID-19 pandemic, restrictions in roles may have also influenced who we were successful in recruiting, and thus the findings. Our paper provides insight through understanding the perceptions of stakeholders and describing what stakeholders perceive to be the challenges and opportunities in the use of digital primary care services and provision of digital facilitation in general practices. Future work may be needed to focus in more depth on single groups of stakeholders with theory generating interviews to develop greater insight into how decisions are made in relation to digital facilitation.^35^

### Implications for policy, practice, and research

As noted, our findings point to the need for greater clarity about the responsibility for the delivery of digital facilitation in primary care at local, regional and national levels. At present, responsibility appears to be assumed to be the role of “others”, and therefore actioning is ad hoc and poorly coordinated. NHS England have recommended practical steps and resources for supporting digital inclusion of patients and staff locally.^26^ These include digital skills training, introducing digital health champions within organisations, intergenerational mentoring from younger people, and raising awareness of digital support available. However, study participants were unable to confirm that this was occurring on the ground within primary care. Developing and implementing policy for the provision of digital facilitation in primary care will likely require the collaboration of a wide range of stakeholders including patients and the wider public, primary care staff, commissioners, third sector organisations and digital platform providers.

A framework for digital inclusion, published in 2023, specifies action for staff, regional and national leaders.^43^ It includes recommendations for connectivity and digital literacy and our findings are timely in showing the need to develop a more comprehensive understanding of digital facilitation so that efforts do not remain isolated, that learning can be shared and in showing the relevance of including a broad range of stakeholders. In 2024, NHS England has also made steps to improve support for GPs and local systems for the procurement and commissioning of digital services, and commitments to greater interoperability of digital technology systems and digital tools.^44^

Individual GP practices remain core to the implementation of digital facilitation and our study found that there was considerable appreciation of the challenges faced. There remain several unanswered questions around the roles of staff, training for staff, and who should fund digital facilitation in primary care.^30,33^ Existing frameworks such as the nonadoption, abandonment, scale-up, spread and sustainability (NASSS) framework may offer a theory informed basis for new digital facilitation policies.^45^ For example, by recognising the complexity in requirements of staff, patients and carers as necessary adopters of different digital services in primary care, the digital facilitation needs may be more systematically identified.

Our findings highlight that a wider range of stakeholders should come together to ensure that support is available for practices and for the co-development of effective digital facilitation interventions and subsequent sharing of learning. Our findings have relevance across countries in the UK and more broadly. We already noted efforts to develop digital services in primary care elsewhere^4–6^ with similar challenges such as fragmented online service offerings, interoperability and concerns around equity.^5^

## Conclusion

Digital facilitation offers potential to increase the uptake of online services in primary care. Our study identifies the need for policy to establish clear lines of responsibility, investment in staff time and training, and the development of digital services that work well for various groups of patients and practice staff. A multi-organisational working team involving policy and practice is needed to establish principles for the provision of support for patients and staff in the access and use of digital primary care services in England.

## Supplemental Material

Supplemental Material - Use of digital facilitation to support the use of digital services in general practice in England: An interview study with key stakeholders Supplemental Material for Use of digital facilitation to support the use of digital services in general practice in England: An interview study with key stakeholders by Bethan Mair Treadgold, Rachel Winder, Helen Atherton, Carol Bryce, John Campbell, Christine Marriott, Jenny Newbould, Stephanie Stockwell and Emma Pitchforth in Journal of Health Services Research & Policy
