# Supplementary material for: Use of digital facilitation to support the use of digital services in general practice in England: An interview study with key stakeholders
Source: J Health Serv Res Policy. 2025 Jan 30;30(3):161–70. doi: 10.1177/13558196251316446 (PMC12138138; doi:10.1177/13558196251316446)
Supplement: Supplemental Material - Use of digital facilitation to support the use of digital services in general practice in England: An interview study with key stakeholders [file sj-pdf-1-hsr-10.1177_13558196251316446.pdf]

Journal of Health Services Research & Policy
Use of digital facilitation to support the use of digital services in general practice in England: An interview study with key stakeholders
Bethan Mair Treadgold et al

Online Supplement

Standards for Reporting Qualitative Research (SRQR)*

http://www.equator-network.org/reporting-guidelines/srqr/

Title and abstract

	Page/line no(s).
**Title** - Concise description of the nature and topic of the study Identifying the study as qualitative or indicating the approach (e.g., ethnography, grounded theory) or data collection methods (e.g., interview, focus group) is recommended	✓
**Abstract** - Summary of key elements of the study using the abstract format of the intended publication; typically includes background, purpose, methods, results, and conclusions	✓

Introduction

**Problem formulation** - Description and significance of the problem/phenomenon studied; review of relevant theory and empirical work; problem statement	✓
**Purpose or research question** - Purpose of the study and specific objectives or questions	✓

Methods

**Qualitative approach and research paradigm** - Qualitative approach (e.g., ethnography, grounded theory, case study, phenomenology, narrative research) and guiding theory if appropriate; identifying the research paradigm (e.g., postpositivist, constructivist/ interpretivist) is also recommended; rationale**	✓
**Researcher characteristics and reflexivity** - Researchers' characteristics that may influence the research, including personal attributes, qualifications/experience, relationship with participants, assumptions, and/or presuppositions; potential or actual interaction between researchers' characteristics and the research questions, approach, methods, results, and/or transferability	✓
**Context** - Setting/site and salient contextual factors; rationale**	✓
**Sampling strategy** - How and why research participants, documents, or events were selected; criteria for deciding when no further sampling was necessary (e.g., sampling saturation); rationale**	✓
**Ethical issues pertaining to human subjects** - Documentation of approval by an appropriate ethics review board and participant consent, or explanation for lack thereof; other confidentiality and data security issues	✓

**Data collection methods** - Types of data collected; details of data collection procedures including (as appropriate) start and stop dates of data collection and analysis, iterative process, triangulation of sources/methods, and modification of procedures in response to evolving study findings; rationale**	✓
**Data collection instruments and technologies** - Description of instruments (e.g., interview guides, questionnaires) and devices (e.g., audio recorders) used for data collection; if/how the instrument(s) changed over the course of the study	✓
**Units of study** - Number and relevant characteristics of participants, documents, or events included in the study; level of participation (could be reported in results)	✓
**Data processing** - Methods for processing data prior to and during analysis, including transcription, data entry, data management and security, verification of data integrity, data coding, and anonymization/de-identification of excerpts	✓
**Data analysis** - Process by which inferences, themes, etc., were identified and developed, including the researchers involved in data analysis; usually references a specific paradigm or approach; rationale**	✓
**Techniques to enhance trustworthiness** - Techniques to enhance trustworthiness and credibility of data analysis (e.g., member checking, audit trail, triangulation); rationale**	✓

Results/findings

**Synthesis and interpretation** - Main findings (e.g., interpretations, inferences, and themes); might include development of a theory or model, or integration with prior research or theory	✓
**Links to empirical data** - Evidence (e.g., quotes, field notes, text excerpts, photographs) to substantiate analytic findings	✓

Discussion

**Integration with prior work, implications, transferability, and contribution(s) to the field -** Short summary of main findings; explanation of how findings and conclusions connect to, support, elaborate on, or challenge conclusions of earlier scholarship; discussion of scope of application/generalizability; identification of unique contribution(s) to scholarship in a discipline or field	✓
**Limitations** - Trustworthiness and limitations of findings	✓

Other

**Conflicts of interest** - Potential sources of influence or perceived influence on study conduct and conclusions; how these were managed	✓

**Funding** - Sources of funding and other support; role of funders in data collection, interpretation, and reporting	✓

*The authors created the SRQR by searching the literature to identify guidelines, reporting standards, and critical appraisal criteria for qualitative research; reviewing the reference lists of retrieved sources; and contacting experts to gain feedback. The SRQR aims to improve the transparency of all aspects of qualitative research by providing clear standards for reporting qualitative research.

**The rationale should briefly discuss the justification for choosing that theory, approach, method, or technique rather than other options available, the assumptions and limitations implicit in those choices, and how those choices influence study conclusions and transferability. As appropriate, the rationale for several items might be discussed together.

Reference:

O'Brien BC, Harris IB, Beckman TJ, Reed DA, Cook DA. **Standards for reporting qualitative research: a synthesis of recommendations.** *Academic Medicine*, Vol. 89, No. 9 / Sept 2014
DOI: 10.1097/ACM.0000000000000388

Topic Guide: Stakeholder Interviews

Study title: Di-Facto: Digital Facilitation in Primary Care

Introduction

- Thank you for taking the time to talk to me today and offering to take part in this study.
- I would like first to outline the study so that you are able to decide whether you wish to proceed further
    - The project is about digital facilitation, which is the range of processes, procedures, and personnel which seeks to support NHS patients and carers of patients in their uptake and use of online services in primary care settings
    - This study aims to understand more about digital facilitation through conducting case studies in general practices to find out about the views and experiences of patients and staff, and interviews with people who may have a wider role in decision making about digital facilitation or relevant experience surrounding supporting the use of online services in primary care settings such as yourself
    - The interview may last between 30 to 60 minutes depending on your preference and capacity today – can I check how long you have today?
- You have kindly signed the consent form ahead of the meeting today. Can I check that you are happy with what you have completed on the form and happy to continue with the interview?
- I have a list of topics that I would like to address for the research but hope that it will flow like a conversation where you feel comfortable to raise any related issues.
- I might just take a few notes in case I want to come back to something later on.
- Just to finally check that you are happy for the interview to be recorded? Although we are using the video link, we will only capture the audio recording

[Questions to be framed to role – e.g. if at national level, focus on national agendas or if local/linked to participating practices, talk more of local area. The questions are indicative of the range of questions that could be covered. The interviews will also ask, where relevant, whether views are those of the individual participant or representative of their organisation]

1) Participant's current role

These questions will seek to understand more about your experience and role in relation to online services in primary care.

- Can you tell me what your current role/job is and what that involves?
- What responsibilities or interest do you have with respect to digital services in primary care? (prompt: can you say a bit more about where relevant)
- Have you had any previous jobs/roles relevant to understanding the use of digital services in primary care? Can you say a bit more?

2) Digital services in primary care

These questions will attempt to understand how you feel about current and changing use of digital services in primary care, the drivers for this and what may be the priority for increasing use of digital services.

- Can you tell me how you feel about the current use of digital services in primary care? How do you feel this has changed over time?
- What are the priorities within your role for increasing use of digital services in primary care? Why is this important?
- What are the drivers for trying to increase the use of digital services in primary care? (prompt: pre/post COVID-19; specific policies/agendas; professional drivers; patient drivers; resources) Which of these do you feel are most important just now/in your areas? Why?

3) Increasing uptake for digital services in primary care

In this section we are interested in views about what is being done to support patients and carers access to use online services or to support staff to help patients and what else you think could be done. This will include a discussion of the challenges that you think general practices face.

We have been interested in this study in understanding different models of 'digital facilitation' where general practices take specific steps to try to support patients, carers or staff to use online services.

- What do you feel are the main challenges for patients and carers in using online services in primary care?
- For what groups of patients and carers do you think it is most challenging to use commonly available online services in primary care? (prompt: particular challenges/groups)

Policy level (as appropriate)

- Can you tell me what you know about what is currently done at a policy level to help staff, patients and carers access online services in primary care? Can you tell me a bit more about these (status of policy, target, aims)
- In your view, how successful have these policies been? Can you say a bit more? (prompts: from what perspective; evidence to support)

On the ground

- Practically, what do you know that general practices are doing to increase and support the uptake of online services? Can you talk about that a bit more/give examples? How effective do you think these efforts are? Why?
- Who has responsibility for ensuring increased uptake of online services in primary care and that particular group of patients and carers are not disadvantaged or excluded (prompts: responsibility at different levels – e.g. staff, practice, PCN, CCG, national)
- Who else do you think has an important role in trying to help to increase uptake of online services in primary care? Why?
- What do you think are the most significant challenges in trying to increase and support the uptake of online services? (prompts: equity, digital exclusion, types of services, COVID-19)
- What can be done to try to meet these challenges? Is there any change that you know of in relation to your role/wider policy that may impact on the uptake of online services in the future?

4) Future

This section aims to concentrate more on what can be done in the future (near/medium term).

- What would be your ideal vision for how online primary care services are used in the future? (E.g. 2/5 years' time) How confident are you that we will be in that position?

- If you could prioritise actions or resources to increase the use of online services what would you recommend? Why?
- What are the opportunities for change at these different levels we have discussed? (prompts: what needs to be done, what are barriers/facilitators around this)
- What will be important for future policy to address and consider in this area? Are there sufficient policy measures to address the concerns and challenges you have raised? Can you say a bit more? (prompts: on issues raised in previous questions, reasons for confidence or not, challenges for policy)

5) Tying up: other considerations

This section explores aspects of digital facilitation that we have not thought of or discussed so far.

- In talking about supporting patients and carers to make use of online services in primary care we have used the term ‘digital facilitation’ in our project which we define as: the range of processes, procedures and personnel which seeks to support NHS patients (or their carers) in their uptake and use of online services. Do you think digital facilitation a useful term? Do you have specific terms you use? Is there anything important missing from our definition?
    - Has this made you think of any other examples that we have not already discussed?
    - From our conversation so far, is there anything else that you think would be important for us to understand? Can you say a bit more?
    - In relation to national or regional actors and practice around digital facilitation is there anyone who you would feel is important to talk to? Why do you recommend?

This study is funded by the National Institute for Health Research (NIHR) [Health Services and Delivery Research Programme 128268]. The views expressed are those of the author(s) and not necessarily those of the NIHR or the Department of Health and Social Care.

Thank you very much for your time today.
